# Oligoprogressive renal cell carcinoma: What is the role of surgery?

**DOI:** 10.1111/bju.70281

**Published:** 2026-04-13

**Authors:** Chiara Re, William H.J. Ince, Leo Bickley, James P. Blackmur, Mouhamad H. Ismail, Justicia Kyeremeh, Teele Kuusk, Eva Mendes Serrao, James O. Jones, Grant D. Stewart

**Affiliations:** ^1^ Department of Surgery University of Cambridge, Cambridge Biomedical Centre Cambridge UK; ^2^ CRUK Cambridge Centre University of Cambridge Cambridge UK; ^3^ IRCCS San Raffaele Scientific Institute, Division of Experimental Oncology/Unit of Urology Urological Research Institute (URI) Milan Italy; ^4^ Department of Oncology Cambridge University Hospitals NHS Foundation Trust Cambridge UK; ^5^ Department of Medicine Cambridge University Hospitals NHS Foundation Trust Cambridge UK; ^6^ Department of Urology Western General Hospital Edinburgh UK; ^7^ Institute of Genetics and Cancer, Western General Hospital University of Edinburgh Edinburgh UK; ^8^ Department of Radiology Cambridge University Hospitals NHS Foundation Trust Cambridge UK

**Keywords:** metastatic kidney cancer, renal cell carcinoma, oligoprogression, oligorecurrence, metastasis‐directed therapy, local treatment

## Abstract

**Objective:**

To provide an overview of the biological mechanism and pattern of oligoprogression in renal cell carcinoma (RCC) and the most updated role of surgery in this setting, highlighting scientific gaps and informing future implications.

**Methods:**

A non‐systematic search of PubMed/MEDLINE was performed in August 2025 including guidelines, reviews, and original studies. Additional sources were identified through manual reference screening.

**Results:**

Oligoprogression in RCC likely arises from clonal evolution dynamics, with spatially confined subclone mutations and microenvironmental niches yielding differential responses to treatment. Complete metastasectomy may offer a survival benefit, spare patients toxic side effects of systemic therapy, and offers the possibility of histopathological and molecular characterisation of residual disease. Surgery is also particularly important in delayed cytoreductive nephrectomy as the site of oligoprogression. However, this must be weighed against non‐negligible surgical morbidity and complication rates. International guidelines recognise the role of surgical resection in the metastatic setting but emphasise low evidence levels and careful patient selection requirements.

**Conclusion:**

Limited evidence derived from the oligometastatic context constrains optimal patient selection and timing strategies in oligoprogressive RCC. Following the establishment of consensus over definitions and research gaps, future research integrating molecular biomarkers, novel imaging tracers, and multicentre collaborative studies will be essential to develop evidence‐based surgical management protocols for this increasingly common clinical scenario.

**Abbreviations**
(d)CN: (deferred) cytoreductive nephrectomy
ctDNA: circulating tumour DNA
DFS: disease‐free survival
HR: hazard ratio
ICI: immune checkpoint inhibitor
MDT: multidisciplinary team
MDTx: metastasis‐directed therapy
NED: no evaluable disease
OAR: organ‐at‐risk
OS: overall survival
PD‐L1: programmed death‐ligand 1
PFS: progression‐free survival
RT: radiotherapy
SABR: stereotactic ablative body RT
RECIST: Response Evaluation Criteria in Solid Tumours
RT: radiotherapy
TKI: tyrosine kinase inhibitor
TME: tumour microenvironment

## Introduction

Since the approval of the first tyrosine kinase inhibitor (TKI) in 2005, the therapeutic landscape of RCC has undergone a profound transformation [1]. Patients with metastatic RCC now have access to a plethora of novel systemic agents, including TKIs and immune checkpoint inhibitors (ICIs), either alone or in combination, which have led to significant improvements in survival outcomes. Despite these advances, complete remission under systemic treatment is only observed in a minority of cases [2], and disease progression often occurs in a limited number of metastatic sites—a phenomenon defined as oligoprogression [3]. In this scenario, the aforementioned therapies remain viable options; however, each successive line of treatment is typically associated with reduced benefits, characterised by shorter disease control intervals and cumulative toxicity [4]. Oligoprogressive disease thus presents a unique therapeutic opportunity where metastasis‐directed therapy (MDTx; i.e., surgery, ablation or radiotherapy [RT]) may play a pivotal role. The rationale of adding local treatment is that resistant lesions may respond to targeted local therapy, while other sites remain sensitive to ongoing systemic therapy. This approach can avoid premature escalation or discontinuation of systemic treatments when alternative therapeutic options may be limited [5]. Furthermore, this strategy may facilitate achievement of no evaluable disease (NED) status and has demonstrated improved overall survival (OS) in carefully selected patients [6, 7]. Within this therapeutic paradigm, while stereotactic ablative body RT (SABR) has gained considerable prominence [8], the role of surgical resection is unclear. Evidence supporting metastasectomy in this setting is inconsistent, with limited retrospective series and no prospective randomised trials addressing its efficacy and safety [9, 10]. Given these gaps, we aimed to reframe oligoprogressive RCC as a unique biological and therapeutic entity, critically evaluate the contemporary role of surgical intervention relative to other local therapies, and highlight research priorities necessary to develop evidence‐based management strategies.

## Methods

### Literature Search

We performed a non‐systematic, narrative review to summarise current evidence on oligoprogressive RCC with a focus on surgical management. A PubMed/MEDLINE comprehensive search was conducted to identify relevant literature, including scientific guidelines, original studies and review articles on the topic. Keywords included ‘renal cell carcinoma’, ‘metastasis’, ‘oligoprogressive’, ‘oligometastatic’, ‘surgical management’, ‘cytoreductive nephrectomy’, ‘local treatment’, ‘metastasis‐directed therapy’, ‘metastasectomy’ and ‘systemic therapy’ along with free‐text or related and derivative terms. The search was conducted in August 2025. To focus on the most relevant and contemporary evidence, we initially included articles published over the past 20 years. Subsequently, additional relevant manuscripts were identified through a manual search of the reference lists of the retrieved articles. The review provides an overview of the biological mechanism and pattern of oligoprogression in RCC and the most updated role of surgery in this setting, highlighting scientific gaps and informing future implications.

### Definitions

In 1995, Hellman and Weichselbaum [11] introduced the concept of ‘oligometastatic disease’ —an intermediate state between localised and widely disseminated cancer—characterised by a limited number of metastatic lesions, typically five or fewer. This framework views metastatic potential as a spectrum, suggesting that some patients with a restricted number of metastases may achieve durable disease control with local treatment. With the increasing efficacy of modern systemic therapies, ‘oligoprogression’ has emerged as a related but distinct clinical entity, defined as progression in a limited number of lesions while other disease sites remain controlled under ongoing systemic therapy [12]. To address this scenario, the European Society for Radiotherapy and Oncology (ESTRO) and the European Organisation for Research and Treatment of Cancer (EORTC) proposed a classification system identifying two main patterns [3]: progression in a few metastatic sites (‘oligoprogressive metastases’) and progression of the primary tumour with stable metastases (‘oligoprogressive primary’). A functional subclassification has also been introduced based on timing and biological behaviour: ‘oligorecurrence’, referring to limited metastatic relapse during a treatment‐free interval, and ‘oligopersistence’, describing stable residual oligometastatic disease following systemic therapy. Ongoing refinement of these definitions reflects the growing complexity of disease management, particularly in RCC, where precise terminology is increasingly important. For example, it remains debated whether limited metastatic disease developing shortly after adjuvant pembrolizumab should be considered oligoprogression or oligorecurrence. A practical vocabulary of technical terminology is reported in Table S1.

## Results

### Biological Mechanism of Oligoprogression

Oligoprogression in RCC can be understood through tumour evolution: subclones with distinct mutations in different stromal and immune niches may have variable fitness under systemic therapy. Multi‐region sequencing of primary and metastatic tumours shows that intra‐tumour heterogeneity is common [13], and specific driver mutations are linked to different clinical trajectories [14]. For example, early BAP1 mutation drives chromosomal instability and clonal sweep, resulting in low heterogeneity and aggressive polymetastatic disease. In contrast, PBRM1 followed by SETD2 mutations create high heterogeneity and a more indolent course, with gradual emergence of oligometastatic disease. In these highly branched phylogenies, competition between subclones (clonal interference) can slow progression [14, 15], allowing therapy‐resistant subclones to remain confined to certain sites [16]. The ADAPTeR study (ClinicalTrials.gov identifier: NCT02446860) illustrated this, finding a resistant subclone only in treatment‐refractory metastases. The tumour microenvironment (TME) further shapes treatment response [17]. Transcriptomic analysis of 823 RCC samples from the IMmotion151 trial (NCT02420821) [18] identified seven molecular clusters associated with variable anti‐angiogenic and ICI response. NAXIVA (NCT03494816) multi‐omics data confirmed that angiogenic and immune signatures predict response and vary by organ site [19]. For example, the IMmotion151 angiogenic/stromal cluster was enriched in lung, skin, endocrine, bone, and gastro‐intestinal metastases, but not in liver, lymph node, or brain [20]. Pancreatic metastases, markers of indolent disease, exhibit high angiogenic signals, low effector T cells, sensitivity to anti‐angiogenics, but ICI resistance [21].

These biological insights have the primary aim of guiding surgical decision‐making. Oligoprogressive lesions are more likely to represent spatially confined, treatment‐resistant subclones, where progression kinetics are slow, and new lesions do not rapidly emerge. In this context, surgical resection may eradicate resistant clones before wider dissemination. Conversely, rapid multifocal progression suggests early polymetastatic escape and argues against aggressive local intervention.

### Guidelines Recommendations

The European Association of Urology (EAU) [22] and AUA [23] guidelines recognise a role for metastasectomy in the management of metastatic RCC, particularly in patients with low metastatic burden and good performance status, provided that complete resection is achievable. However, both guidelines consistently underline that the level of evidence remains low, with recommendations largely based on retrospective data. The European Society of Medical Oncology (ESMO) [24] guidelines state that MDTx, including metastasectomy, thermal ablation, and SABR, can be considered in carefully selected patients following multidisciplinary discussion. Notably, it should be avoided in patients with a high burden of metastases, short interval to recurrence or aggressive disease. Similarly, the National Comprehensive Cancer Network (NCCN) Guidelines [25] recommend considering metastasectomy (or alternative local ablative techniques) in two main scenarios: (i) patients presenting with primary RCC and synchronous oligometastatic sites, or (ii) patients who develop oligometastases after a prolonged disease‐free interval following nephrectomy (Table 1 [22, 23, 24, 25]). Importantly, no guidelines refer specifically to oligoprogression, but instead deal with oligometastasis in general, despite clear differences in biology as outlined above. Several key issues, such as the optimal timing of systemic treatment and the best strategy for integrating systemic and local approaches, remain unresolved. Furthermore, no definitive evidence or consensus supports the preferential use of either surgery or SABR in the management of oligometastatic disease, and oligoprogressive status represents an even more significant knowledge gap.

**Table 1:** Local treatment recommendations for patients with metastatic RCC according to international guidelines.

European Association of Urology (EAU) Guidelines [22]	To control local symptoms, offer ablative therapy, including metastasectomy, to patients with metastatic disease and favourable disease factors and in whom complete rection is achievable
AUA guidelines [23]	Surgical resection or ablative therapies should be considered in select patients with isolated or oligometastatic disease
European Society for Medical Oncology (ESMO) Clinical Practice Guidelines [24]	Metastasectomy, thermal ablation, stereotactic radiosurgery, SBRT, CyberKnife RT and hypofractionated RT can be considered for selected patients with low metastatic burden after MDT review. Typically, these treatments focus on a single site of disease. These strategies should be avoided in patients with a high burden of metastases, short interval to recurrence or aggressive disease
National Comprehensive Cancer Network (NCCN) Guidelines [25]	In addition, the small subset of patients with potentially surgically resectable primary RCC and oligometastatic sites are candidates for nephrectomy and management of metastases by surgical metastasectomy or with ablative techniques for selected patients who are not candidates for metastasectomy Candidates include patients who: (i) initially present with primary RCC and oligometastatic sites; or (ii) develop oligometastases after a prolonged disease‐free interval from nephrectomy Oligometastatic sites that are amenable to this approach include the lung, bone, and brain

### Surgical Metastasectomy Outcomes

#### Oligometastatic Setting

Surgical resection for metastatic RCC was historically performed in the absence of effective systemic therapies, mainly to achieve local control in selected patients with good performance status or for symptom relief. It is historically well known that the site of metastasis significantly influences prognosis and treatment outcomes [26]. Metastases confined to the lung, lymph nodes, or soft tissue are generally considered favourable sites, often associated with better response to systemic therapy and longer survival, whereas those involving the liver, bone, or brain are regarded as unfavourable due to their association with more aggressive disease biology, reduced therapeutic efficacy, and poorer overall prognosis [27].

Currently, large prospective trials specifically treating oligoprogressive cohorts are lacking, and current knowledge derived from systematic reviews and meta‐analyses of heterogeneous retrospective series in oligometastatic populations, often predate modern therapies. This limitation is evident in the available literature: Zaid et al. [28] analysed eight comparative studies comprising 2267 patients with metastatic RCC, 958 of whom underwent metastasectomy, revealing a median OS of 36.5–142 months, compared to 8.4–27 months for those who did not undergo surgery; however, only two of these studies included patients treated after 2008. Similarly, Ouzaid et al. [7] reviewed 56 retrospective studies suggested that metastasectomy in patients with oligometastatic RCC, when integrated into multimodal management, may reduce treatment toxicities and preserve quality of life without compromising survival, though these findings are also based on many studies that have been conducted before the era of TKIs and ICIs.

In this scenario, several studies emphasised the need for complete resection to achieve a survival advantage compared with incomplete or absent metastasectomy [29], a principle still endorsed by current guidelines [22, 23]. In a Mayo Clinic oligometastatic series, complete metastasectomy was associated with superior 2‐year OS (84% vs 54%) and reduced risk of RCC‐related death (hazard ratio [HR] 0.47, 95% CI 0.34–0.65; *P* < 0.001) [30].

When evaluating metastasectomy outcomes, it is important to recognise that surgical candidacy is influenced by multiple factors, including lesion operability and resectability, patient comorbidities, the risk of interrupting or delaying systemic therapy, and the expected morbidity of surgery. A population‐based study of patients with oligometastatic RCC from the National Inpatient Sample database [31] suggested an overall complication rate of 45.7%, with major complications (Clavien–Dindo III–V) constituting 27.5%. Similarly, Palumbo et al. [32] evaluated 3654 patients with oligometastatic RCC treated with metastasectomy after targeted therapy within the Surveillance, Epidemiology and End Results (SEER) database and reported complications and in‐hospital mortality of 55.0% and 4.6%, respectively. Furthermore, Lyon et al. [33] investigated that factors such as advanced age, multiple sites of resection, and pancreatic resection were associated with major complications (*P* < 0.05). It is important to note that the complication rates reported from oligometastatic population‐based datasets may not accurately reflect outcomes in carefully selected oligoprogressive patients treated at experienced, high‐volume centres. Appropriate context regarding patient selection, systemic treatment and experience of the centre is therefore required.

An important question following MDTx for the outcomes oligometastatic disease is the role of subsequent systemic therapy. The evidence base in this setting has notably expanded in recent years, with the inclusion of small subgroups of patients with M1 NED in two pivotal randomised phase III trials of adjuvant immunotherapy: KEYNOTE‐564 (adjuvant pembrolizumab vs placebo; NCT03142334) [34] and RAMPART (adjuvant durvalumab/tremelimumab vs observation; NCT03288532) [35]. Both trials showed a significant disease‐free survival (DFS) benefit in the intention‐to‐treat population, with KEYNOTE‐564 having also shown a significant OS benefit. Exploratory subgroup analysis for the M1 NED subgroup has been published for KEYNOTE‐564 and, with the caveats of limited sample size and unpowered subgroup analysis, this showed a HR for DFS of 0.72 (95% CI 0.59–0.87) favouring adjuvant pembrolizumab [34]. These data support the concept that adjuvant immunotherapy can be of benefit in patients who have undergone metastasectomy [34].

#### Oligoprogressive Setting

In the oligoprogressive setting, only three case studies (Level 3 evidence) have been published on surgical resection, highlighting the paucity of evidence in this clinical scenario. Santini et al. [9] analysed 55 patients, who had experienced oligoprogression under TKI therapy, with progression occurring principally in the lung (27.3%), bone (18.2%) and the contralateral kidney (14.5%). Among other local treatments, 25 patients (45.5%) underwent surgical removal of metastatic lesions. Overall, for all the local treatment performed (RT, ablation, surgery), the reported median progression‐free survival (PFS) and OS, were 14 months (95% CI 6.9–21 months) and 37 months (95% CI 25.2–48.7 months), respectively [9]. Le et al. [10] evaluated 10 patients with thoracic progression under ICI (40%) or TKI (50%), reporting a median PFS of 10 months (95% CI 2–29 months) and OS of 24 months (95% CI 2–73 months). More recently, Hoffer et al. [36] reported data on 315 patients, of whom 51.7% received a local treatment. The lung (57.7%) and lymph nodes (33.7%) were the predominant oligoprogression sites, followed by bone (27.6%) and liver (12.3%). In all, 60 patients (41.7%) underwent surgery, revealing a median OS of 49.6 months (95% CI 34.7–64.5 months) [36] (Table 2 [9, 10, 36]). Critically, across these published studies, data on surgical resection for oligoprogressive disease under ICIs exist for only eight patients (four in Le et al. [10] and four in Hoffer et al. [36]). This represents a striking evidence gap, particularly given the standard‐of‐care use of ICIs, in contemporary oncology practice. Consequently, clinical decision‐making must currently rely on extrapolation from general oligometastatic principles, underscoring the urgent need for prospective studies specifically addressing this population.

**Table 2:** Evidence for surgical metastasectomy in oligoprogressive RCC.

References	First‐line SACT, *n* (%)	Patient number	Oligoprogression site, *n* (%)	Local treatment type, *n* (%)	PFS, months (95% CI)	OS, months (95% CI)
Santini et al. (2017) [9]	Sunitinb 48 (87.3) Pazopanib 5 (9.1) Sorafenib 2 (3.6)	55	Lung 15 (27.3) Bone 10 (18.2) Contralateral kidney 8 (14.5) Liver 4 (7.3) Brain 4 (7.3) Others 14 (25.4)	Ablative techniques 5 (9.1) Surgery 25 (45.5) RT 25 (45.5)	14 (6.9–21)	37 (25.2–48.7)
Le et al. (2023) [10]	Axitinib 1 (10) Nivolumab 4 (40) Sorafenib 2 (20) Everolimus 1 (10) Sunitinib 2 (20)	10	Lung 8 (80) Lymph nodes 2 (20) Chest wall 1 (10)	Surgery 10 (100)	10 (2–29)	24 (2–73)
Hoffer et al. (2025) [36]	VEGF 224 (71.1) mTOR 13 (4.1) Cytokine 38 (12.1) Cytokine + chemo 24 (7.6) ICI 4 (1.3) Chemo 3 (0.9) Others 9 (2.9)	315 (163 [51.7%] local treatment)	Lung 94 (57.7) Lymph nodes, 55 (33.7) Bones 45 (27.6) Liver 20 (12.3) Soft tissue 15 (9.2) Contralateral kidney 12 (7.4) Local recurrence 9 (5.5) Brain 3 (1.8)	SABR 80 (49.1) Surgery 68 (41.7) RFA 4 (2.5) SIRT 2 (1.2) Others 9 (5.5)	NA	49.6 (34.7–64.5)

Chemo, chemotherapy; mTOR, mammalian target of rapamycin; RFA, radiofrequency ablation; SACT, systemic anti‐cancer therapy; SIRT, selective internal RT.

### The Role of Cytoreductive Nephrectomy (CN)

Cytoreductive nephrectomy may play a pivotal role in the treatment of oligoprogressive disease, when the primary renal cancer is no longer controlled by ongoing systemic therapy. In this context it is crucial to correctly differentiate oligoprogressive (primary tumour increasing in size) from oligopersistence (primary tumour not increasing in size). The biological rationale for CN in metastatic RCC has been supported by findings from the TRACERx Renal study (NCT03226886) [14]. Renal cancer is highly heterogeneous, with distinct genetic mutations across tumour regions and metastases. Removing the primary tumour can reduce overall tumour burden and genetic complexity, limiting the emergence of resistant clones that could drive further disease progression. CN also eliminates the primary tumour as a source of ongoing metastatic seeding, potentially slowing progression. Additionally, by removing an immunosuppressive tumour source, CN may enhance systemic immune response and improve the efficacy of immunotherapy. CN may also alleviate local symptoms (e.g., pain, bleeding) [6]. Retrospective series and multicentre studies have consistently reported that deferred CN (dCN) is both safe and technically feasible in appropriately selected patients, with outcomes suggesting a therapeutic advantage when performed after response to systemic therapy [37, 38]. Careful patient selection is crucial to maximise the potential benefits of delayed surgery, reduce perioperative risks and improve oncological outcomes. While these findings support the role of dCN, surgery after ICI exposure can be technically challenging due to desmoplastic and inflammatory reactions, highlighting the importance of centralising care in expert centres [37]. However, prospective validation is awaited. Ongoing randomised trials, including NORDIC‐SUN (NCT03977571), PROBE (NCT04510597), Cyto‐KIK (NCT04322955) and PrimerX (NCT05941169), are testing the effect of dCN in patients with metastatic RCC receiving contemporary treatment, and are expected to provide more definitive guidance on the timing and role of CN in the current treatment landscape.

### The Role of SABR

Stereotactic ablative body RT has become the dominant non‐surgical MDTx in oligoprogressive RCC because kidney cancer, although historically labelled radioresistant, demonstrates high‐dose per fraction sensitivity when hypofractionation is used [8]. Contemporary series and meta‐analyses report 1–2 year local control rates—defined as absence of radiographic progression within the treated field—of approximately 80–95%. Ablative schedules commonly include 24–30 Gy in three fractions, 30–40 Gy in five fractions, or a single fraction of 18–24 Gy, selected according to lesion size and organ‐at‐risk (OAR) constraints; OAR constraints refer to dose limits for adjacent critical structures, such as bowel loops, spinal cord, stomach, or major vessels, beyond which the risk of toxicity becomes unacceptable [8, 39]. From a mechanistic perspective, the rationale for combining SABR with immunotherapy in oligoprogressive RCC is supported by preclinical and translational insights. High‐dose per fraction RT induces immunogenic cell death, characterised by calreticulin exposure, ATP release and high mobility group box 1 (HMGB1) signalling, which facilitate dendritic cell recruitment and tumour antigen presentation [40]. Radiation also generates cytosolic double‐stranded DNA, activating the cyclic GMP–AMP synthase–stimulator of interferon genes (cGAS–STING) pathway, leading to type I interferon release and T‐cell priming [41, 42]. These effects increase major histocompatibility complex class I (MHC‐I) expression and programmed death‐ligand 1 (PD‐L1) upregulation on tumour cells, potentially sensitising them to programmed death 1 (PD‐1)/PD‐L1 blockade [43]. However, SABR efficacy is size‐dependent, and OAR constraints may preclude delivery of ablative doses for large or previously irradiated lesions. In such cases, surgery may offer more reliable local control when resection is technically feasible. Practical integration with systemic therapy also drives modality choice: vascular endothelial growth factor (VEGF)‐pathway TKIs can potentiate mucosal/skin toxicity and impair healing, and most centres pause TKIs around SABR to mitigate rare but reported gastrointestinal complications in abdominal fields. By contrast, SABR delivered during ICIs is generally feasible with acceptable toxicity. Early prospective efforts explored whether SABR can potentiate ICIs in metastatic RCC. The RADVAX RCC trial (NCT03065179), a single‐arm phase I/II study combining SABR with nivolumab and ipilimumab, reported an overall response rate of 56% among 25 patients, numerically higher than historical ICI alone. Similarly, the NIVES trial (NCT03469713) investigated SABR followed by nivolumab monotherapy in 69 previously treated patients; While SABR was safe and feasible, the study did not meet its primary endpoint, reporting an overall response rate of 18% [44]. Despite these limitations, both studies demonstrate that SABR can be integrated with ICIs in RCC with acceptable toxicity, and they highlight the biological rationale for pursuing this strategy in the oligoprogressive setting. Phase III randomised trials of SABR in patients with metastatic RCC treated with ICIs, SAMURAI (NCT05327686) and CYTOSHRINK (NCT04090710), are ongoing.

### Decision‐Making Between Metastasectomy and SABR

Understanding the benefits and limitations of metastasectomy and SABR is essential to guide patient‐centred decision‐making within a multidisciplinary setting. In selected cases, a combination of surgery and SABR may be appropriate, such as symptomatic brain metastases or bone metastases with pathological fractures, but in most situations, a choice between the two modalities is required. Complete surgical removal of a metastatic lesion allows for potential eradication with clear margins and provides tissue for histopathological assessment. This enables enabling diagnostic confirmation, biological characterisation, and removal of treatment‐resistant clones [45]. Surgery also allows assessment of pathological response, which may guide subsequent systemic therapy [46]. Surgery may be preferable for symptomatic lesions or for metastases located where RT is limited by dose constraints (e.g., bowel proximity or previously irradiated sites). This consideration is particularly relevant in oligoprogressive patients receiving systemic therapy, where combined toxicity with TKIs or ICIs may be exacerbated [47]. Practical factors also influence modality choice: while surgery is widely available, SABR requires specialised equipment and expertise, which may limit access in some centre (Table 3) [8, 39]. Nevertheless, patient selection for metastasectomy is critical. Surgery is generally reserved for younger patients with good performance status, limited comorbidities, low disease burden (typically one to three lesions), and technically resectable disease. Risks related to anaesthesia, postoperative recovery, treatment interruption, and the potential need for repeated interventions must be carefully weighed. Although both approaches can achieve local control, their comparative effectiveness across anatomical sites and patient populations remains poorly defined, representing an important evidence gap [48].

**Table 3:** Advantages and limitations of metastasectomy and SABR.

	Metastasectomy	SABR
Indications	Complete surgical removal achievable with minimal morbidity in isolated, accessible lesions. Symptomatic progression (e.g., bleeding, obstruction, pain)	Multiple or anatomically challenging lesions in patients unfit for surgery
Candidate	Patients with good performance status, low comorbidity burden, and acceptable surgical risk	Patients with significant comorbidities, advanced age, or high surgical risk; lesions in critical anatomical locations
Lesions characteristics	Single or limited number of metastases (typically ≤3); straightforward sites (e.g., wedge lung resection, adrenalectomy)	Multiple lesions or lesions in surgically challenging locations
Advantages	Pathological confirmation and complete lesion removal Definitive local control with established durability Clear surveillance imaging post‐resection Size‐independent efficacy Established long‐term outcomes data	Minimally invasive with lower acute morbidity Simultaneous treatment of multiple sites Suitable for anatomically challenging locations Shorter recovery period with minimal systemic therapy interruption Outpatient procedure Repeatable
Limitations	Surgical morbidity Potential delays in systemic therapy resumption Limited to surgically accessible sites Requires adequate performance status and organ function	Size‐dependent efficacy (optimal for lesions <5 cm) Potential radiation toxicity to adjacent organs Uncertainty regarding long‐term durability Risk of incomplete tumour eradication Limited long‐term follow‐up data
Duration	Single procedure	Multiple treatment (lesion/s dependent)
Retreatment	Limited by prior surgeries	Possible for local recurrence with dose constraints*

* 24–30 Gy delivered in three fractions, 30–40 Gy in five fractions, or a single fraction of 18–24 Gy.

### Other Ablative Treatment Options

Ablative local treatments are also increasingly used for local recurrences and metastatic RCC, demonstrating high technical success and an acceptable safety profile. Welch et al. [49] reported effective local control of thermal ablation across multiple metastatic sites, with low rates of technical failure (4.9%), limited local recurrence (8.9%), and a favourable 3‐year OS rate (76%). Similarly, several studies observed comparable efficacy with cryoablation, particularly when combined with systemic therapy, showing favourable OS outcomes and low complication rates [50]. More recently, ablation has also been explored for primary tumours in the metastatic setting, showing OS comparable to CN [51].

### The Importance of a Multidisciplinary Team (MDT)

An individual patient may develop multiple and distinct oligometastatic states over the course of disease, necessitating repeated courses of local and systemic treatment. This clinical heterogeneity makes MDT involvement essential. MDT discussion enables comprehensive evaluation of disease extent, therapeutic options, and treatment sequencing, integrating expertise from medical oncology, radiation oncology, urology, radiology, and organ‐specific surgical subspecialties. Collaboration across disciplines ensures that decisions are tailored to tumour biology, symptom burden, patient comorbidities, and quality‐of‐life considerations [52]. Regular MDT reassessment is also critical, as evolving patterns of progression may alter therapeutic priorities. However, as systemic therapies extend survival and the number of potentially eligible patients increases, the volume of cases requiring MDT review has grown substantially. This expansion has prompted consideration of streamlined MDT models, whereby clearly defined referral criteria, pre‐meeting triage, complex cases prioritisation could optimise workflow and preserve the depth of multidisciplinary input without overburdening existing structures [53] (two clinical cases of oligoprogressive RCC managed by MDT are presented in Appendix S1).

### Research Gaps and Future Directions

This review explored oligoprogression in RCC, analysing biological patterns and focusing on surgical management, drawing insights from the oligometastatic setting. Several research gaps emerge in this field. First, a standardised definition for oligoprogressive RCC is urgently needed to enable consistent patient selection, study design, and interpretation of clinical outcomes. Current studies apply heterogeneous criteria, including variable consideration of the primary tumour and inconsistent requirements for systemic therapy stability, limiting comparability and obscuring true disease prevalence and treatment benefit.

Second, conventional cross‐sectional imaging of the chest, abdomen, and pelvis represents the standard of care; however, it cannot reliably distinguish truly oligoprogressive patients from those likely to develop widespread progression after local therapy. Advanced techniques such as prostate‐specific membrane antigen‐positron emission tomography (PSMA‐PET)/CT or diffusion‐weighted MRI are promising but remain underutilised in metastatic settings due to costs and limited evidence. Additionally, the conventionally used criteria for response assessment, Response Evaluation Criteria in Solid Tumours (RECIST) criteria, fail to distinguish progression patterns [54]. This is particularly problematic for surgical decision‐making, as RECIST cannot differentiate between limited oligoprogressive lesions amenable to metastasectomy and widespread microscopic disease that would render surgery futile, potentially leading to either unnecessary interventions or missed therapeutic opportunities.

Third, the current role of metastasectomy is largely empirical, based on extrapolated data from limited retrospective series. This weak evidence base undermines our understanding of optimal patient selection, timing, and surgical techniques. Fourth, there are no established criteria for identifying surgical candidates. Current selection relies on traditional surgical assessments, including performance status, comorbidities, and technical resectability, rather than tumour biology or treatment response. This approach fails to account for the unique biological characteristics that distinguish oligoprogressive disease from widespread progression. Furthermore, the optimal timing of surgical intervention remains equally uncertain: questions persist about whether surgery should be performed at the first sign of oligoprogression, after a period of observation, or only after systemic therapy failure.

Lastly, designing prospective studies for oligoprogressive RCC surgery faces several challenges. The relatively low incidence of oligoprogressive disease limits patient accrual, requiring multi‐institutional collaboration or extended study periods. The heterogeneity in primary treatment regimens, progression sites, and patient characteristics further complicates study design and interpretation.

Numerous opportunities for future research could significantly advance the field. The prospective registry of longitudinal data, including imaging, blood samples, and pathology in both localised and advanced disease, will be paramount to understanding the molecular characteristics of oligoprogressive disease, using the concepts of the ARTIST_RCC trial (NCT04060537) and ‘Multiomic Analysis of Immunotherapy Features Evidencing Success and Toxicity’ (MANIFEST) trial [55]. Moreover, the development and validation of molecular biomarkers to predict surgical benefit, e.g., kidney injury molecule 1 (KIM‐1) could improve patient selection [55]. Novel imaging tracers like ^89^Zr‐DFO‐girentuximab targeting carbonic anhydrase IX (CAIX) [56] and PSMA [57], may identify those most likely to benefit from local strategies. Furthermore, advance imaging modalities, including radiomics and artificial intelligence‐based analysis [58], could improve the detection and characterisation of oligoprogressive lesions, enabling tailored surgical planning and prognostication. Although circulating tumour (ct)DNA detection in RCC has historically proven to be challenging [59], second‐generation ctDNA assays showed promising results in a recent phase II oligometastasis SABR trial by Tang et al. [60]. In particular, ctDNA at baseline and at the 3‐month follow‐up was an independent prognostic factor for systemic therapy‐free survival. ctDNA could serve as a biomarker to identify patients who would benefit most from MDTx or those who might require earlier intervention with systemic therapy.

Novel surgical approaches, such as minimally invasive techniques and intraoperative imaging, may expand eligibility while reducing morbidity. From the biological standpoint, translational research to better explore TME, molecular landscape and immune composition could inform both resistant mechanisms to therapeutic combinations and surgical decision‐making.

## Conclusion

Oligoprogressive RCC represents a distinct evolutionary and therapeutic state emerging in the era of effective ICI‐based systemic therapy, yet its optimal local management, particularly the role of surgery, remains poorly defined.

In the current era, prolonging disease‐free intervals with delay or discontinuation of systemic therapy are more realistic objectives than cure. In selected patients with metastatic RCC, surgical resection of oligoprogressive lesions during systemic therapy may provide durable disease control while mitigating the cumulative toxicities associated with prolonged systemic treatment. Moreover, it preserves subsequent therapeutic options and enables histopathological and molecular characterisation of resistant clones. Surgery has also a pivotal role in delayed CN if the progression is the primary tumour. While early results suggest potential benefits for carefully selected patients, the lack of robust evidence limits our ability to optimise patient selection, surgical timing, and technique selection. Addressing these research gaps through innovative study designs, molecular characterisation, and collaborative efforts will be essential to establish metastasectomy as an evidence‐based component of oligoprogressive RCC management. Until such data are available patients with RCC with oligoprogressive disease should be advised by a MDT able to provide all suitable options in a considered manner.

## Disclosure of Interests

Grant D. Stewart reports educational grants from AstraZeneca; consultancy fees from Evinova and Qurin; travel expenses from MSD; and leadership roles as clinical lead (urology) for the National Kidney Cancer Audit and topic advisor for the National Institute of Health and Care Excellence kidney cancer guidelines. James O. Jones reports consultancy fees from Evinova. The remaining authors have nothing to disclose.

## Funding

The authors have nothing to report.

## Supporting information


**Table S1** Vocabulary.

## References

[1] U.S. Food & Drug Administration . FDA Approval letter for use of sorafenib in advanced renal cancer.

[2] Thouvenin J , Masson C , Boudier P et al. Complete response in metastatic clear cell renal cell carcinoma patients treated with immune‐checkpoint inhibitors: remission or healing? How to improve patients' outcomes? Cancers (Basel) 2023; 15: 793 36765750 10.3390/cancers15030793 PMC9913235

[3] Guckenberger M , Lievens Y , Bouma S et al. Characterisation and classification of oligometastatic disease: a European Society for Radiotherapy and Oncology and European Organisation for Research and Treatment of Cancer consensus recommendation. Lancet Oncol 2020; 21: e18–e28 31908301 10.1016/S1470-2045(19)30718-1

[4] Tang C , Msaouel P . Charting the path to systemic therapy de‐escalation—oligometastatic kidney cancer as a paradigm. JAMA Oncol 2024; 10: 561–562 38451536 10.1001/jamaoncol.2023.7266

[5] Brown JE , Royle KL , Gregory W et al. Temporary treatment cessation versus continuation of first‐line tyrosine kinase inhibitor in patients with advanced clear cell renal cell carcinoma (STAR): an open‐label, non‐inferiority, randomised, controlled, phase 2/3 trial. Lancet Oncol 2023; 24: 213–227 36796394 10.1016/S1470-2045(22)00793-8

[6] Dason S , Wang SJ , Franceschelli D , Singer EA . Metastasis‐directed therapy in oligometastatic and oligoprogressive renal cell carcinoma. Curr Opin Urol 2025; 35: 194–204 39744755 10.1097/MOU.0000000000001254

[7] Ouzaid I , Capitanio U , Staehler M et al. Surgical Metastasectomy in renal cell carcinoma: a systematic review. Eur Urol Oncol 2019; 2: 141–149 31017089 10.1016/j.euo.2018.08.028

[8] Siva S , Kothari G , Muacevic A et al. Radiotherapy for renal cell carcinoma: renaissance of an overlooked approach. Nat Rev Urol 2017; 14: 549–563 28631740 10.1038/nrurol.2017.87

[9] Santini D , Ratta R , Pantano F et al. Outcome of oligoprogressing metastatic renal cell carcinoma patients treated with locoregional therapy: a multicenter retrospective analysis. Oncotarget 2017; 8: 100708 29246014 10.18632/oncotarget.20022 PMC5725056

[10] Le UT , Passlick B , Schmid S . Surgery for thoracic oligoprogression in metastatic renal cell cancer in the era of new systemic therapies. J Thorac Dis 2023; 15(3): 1133–1141 37065601 10.21037/jtd-22-1120 PMC10089849

[11] Hellman S , Weichselbaum RR . Oligometastases. J Clin Oncol 1995; 13: 8–10 7799047 10.1200/JCO.1995.13.1.8

[12] Niibe Y , Hayakawa K . Oligometastases and oligo‐recurrence: the new era of cancer therapy. Jpn J Clin Oncol 2010; 40: 107–111 20047860 10.1093/jjco/hyp167 PMC2813545

[13] Gerlinger M , Rowan AJ , Horswell S et al. Intratumour heterogeneity and branched evolution revealed by multiregion sequencing. N Engl J Med 2012; 366: 883–892 22397650 10.1056/NEJMoa1113205 PMC4878653

[14] Turajlic S , Xu H , Litchfield K et al. Tracking cancer evolution reveals constrained routes to metastases: TRACERx renal. Cell 2018; 173: 581–594 29656895 10.1016/j.cell.2018.03.057 PMC5938365

[15] Pallikonda HA , Turajlic S . Predicting cancer evolution for patient benefit: renal cell carcinoma paradigm. Biochim Biophys Acta Rev Cancer 2022; 1877: 188759 35835341 10.1016/j.bbcan.2022.188759

[16] Au L , Hatipoglu E , Robert de Massy M et al. Determinants of anti‐PD‐1 response and resistance in clear cell renal cell carcinoma. Cancer Cell 2021; 39: 1497–1518 34715028 10.1016/j.ccell.2021.10.001 PMC8599450

[17] Quail DF , Joyce JA . Microenvironmental regulation of tumor progression and metastasis. Nat Med 2013; 19: 1423–1437 24202395 10.1038/nm.3394 PMC3954707

[18] Motzer RJ , Banchereau R , Hamidi H et al. Molecular subsets in renal cancer determine outcome to checkpoint and angiogenesis blockade. Cancer Cell 2020; 38: 803–817 33157048 10.1016/j.ccell.2020.10.011 PMC8436590

[19] Wray R , Paverd H , Machado I et al. Angiogenic and immune predictors of neoadjuvant axitinib response in renal cell carcinoma with venous tumour thrombus. Nat Commun 2025; 16: 3870 40295487 10.1038/s41467-025-58436-8 PMC12037771

[20] Gulati S , Barata PC , Elliott A et al. Molecular analysis of primary and metastatic sites in patients with renal cell carcinoma. J Clin Invest 2024; 134: e176230 39007269 10.1172/JCI176230 PMC11245151

[21] Singla N , Xie Z , Zhang Z et al. Pancreatic tropism of metastatic renal cell carcinoma. JCI Insight 2020; 5: e134564 32271170 10.1172/jci.insight.134564 PMC7205259

[22] Bex A , Ghanem YA , Albiges L et al. European Association of Urology guidelines on renal cell carcinoma: the 2025 update. Eur Urol 2025; 25: 2838 10.1016/j.eururo.2025.02.020 40118739

[23] Campbell SC , Clark PE , Chang SS , Karam JA , Souter L , Uzzo RG . Renal mass and localized renal cancer: evaluation, management, and follow‐up: AUA guideline: part I. J Urol 2021; 206: 199–208 34115547 10.1097/JU.0000000000001911

[24] Powles T , Albiges L , Bex A , Comperat E et al. Renal cell carcinoma: ESMO clinical practice guideline for diagnosis, treatment and follow‐up. Ann Oncol 2024; 35: 692–706 38788900 10.1016/j.annonc.2024.05.537

[25] Motzer RJ , Jonasch E , Agarwal N et al. NCCN Guidelines Insights: Kidney Cancer, Version 3.2025. 2025

[26] Chandrasekar T , Klaassen Z , Goldberg H , Kulkarni GS , Hamilton RJ , Fleshner NE . Metastatic renal cell carcinoma: patterns and predictors of metastases‐a contemporary population‐based series. Urol Oncol 2017; 35: 661 10.1016/j.urolonc.2017.06.060 28728748

[27] Dudani S , de Velasco G , Wells C et al. Sites of metastasis and survival in metastatic renal cell carcinoma (mRCC): results from the international mRCC database consortium (IMDC). J Clin Oncol 2020; 38: 642

[28] Zaid HB , Parker WP , Safdar NS et al. Outcomes following complete surgical Metastasectomy for patients with metastatic renal cell carcinoma: a systematic review and meta‐analysis. J Urol 2017; 197: 44–49 27473875 10.1016/j.juro.2016.07.079

[29] Alt AL , Boorjian SA , Lohse CM , Costello BA , Leibovich BC , Blute ML . Survival after complete surgical resection of multiple metastases from renal cell carcinoma. Cancer 2011; 117: 2873–2882 21692048 10.1002/cncr.25836

[30] Lyon TD , Thompson RH , Shah PH et al. Complete surgical Metastasectomy of renal cell carcinoma in the post‐cytokine era. J Urol 2020; 203: 275–282 31393812 10.1097/JU.0000000000000488

[31] Meyer CP , Sun M , Karam JA et al. Complications after Metastasectomy for renal cell carcinoma‐a population‐based assessment. Eur Urol 2017; 72: 171–174 28359734 10.1016/j.eururo.2017.03.005

[32] Palumbo C , Pecoraro A , Knipper S et al. Survival and complication rates of Metastasectomy in patients with metastatic renal cell carcinoma treated exclusively with targeted therapy: a combined population‐based analysis. Anticancer Res 2019; 39: 4357–4361 31366530 10.21873/anticanres.13604

[33] Lyon TD , Roussel E , Sharma V et al. International multi‐institutional characterization of the perioperative morbidity of Metastasectomy for renal cell carcinoma. Eur Urol Oncol 2023; 6: 76–83 36509653 10.1016/j.euo.2022.11.003

[34] Choueiri TK , Tomczak P , Park SH et al. Overall survival with adjuvant Pembrolizumab in renal‐cell carcinoma. N Engl J Med 2024; 390: 1359–1371 38631003 10.1056/NEJMoa2312695

[35] Larkin J , Powles T , Frangou E et al. First results from RAMPART: An international phase III randomised‐controlled trial of adjuvant durvalumab monotherapy or combined with tremelimumab for resected primary renal cell carcinoma (RCC). 2025; Available at: https://cslide.ctimeetingtech.com/esmo2025/attendee/confcal/show/session/106

[36] Hoffer S , Eggers H , Fröhlich T et al. Impact of local therapy in metastatic renal cell carcinoma during medical treatment in a retrospective analysis. Sci Rep 2025; 15: 27843 40738926 10.1038/s41598-025-10926-x PMC12310947

[37] Graafland NM , Szabados B , Tanabalan C et al. Surgical safety of deferred cytoreductive nephrectomy following pretreatment with immune checkpoint inhibitor‐based dual combination therapy. Eur Urol Oncol 2022; 5: 373–374 34933813 10.1016/j.euo.2021.11.004

[38] Rioja P , Passarella C , Angel M et al. Impact of deferred cytoreductive nephrectomy in the era of the immunotherapy in metastatic renal cell carcinoma: a multicenter retrospective study. JCO 2025; 43: 502

[39] Correa RJM , Louie AV , Zaorsky NG et al. The emerging role of stereotactic ablative radiotherapy for primary renal cell carcinoma: a systematic review and meta‐analysis. Eur Urol Focus 2019; 5: 958–969 31248849 10.1016/j.euf.2019.06.002

[40] Golden EB , Frances D , Pellicciotta I et al. Radiation fosters dose‐dependent and chemotherapy‐induced immunogenic cell death. Onco Targets Ther 2014; 3: e28518 10.4161/onci.28518 PMC4106151 25071979

[41] Deng L , Liang H , Xu M et al. STING‐dependent cytosolic DNA sensing promotes radiation‐induced type I interferon‐dependent antitumor immunity in immunogenic tumors. Immunity 2014; 41: 843–852 25517616 10.1016/j.immuni.2014.10.019 PMC5155593

[42] Harding SM , Benci JL , Irianto J , Discher DE , Minn AJ , Greenberg RA . Mitotic progression following DNA damage enables pattern recognition within micronuclei. Nature 2017; 548: 466–470 28759889 10.1038/nature23470 PMC5857357

[43] Sharabi AB , Lim M , DeWeese TL , Drake CG . Radiation and checkpoint blockade immunotherapy: radiosensitisation and potential mechanisms of synergy. Lancet Oncol 2015; 16: e498–e509 26433823 10.1016/S1470-2045(15)00007-8

[44] Franceschelli D , Checcucci E , De Luca S et al. Nivolumab after stereotactic body radiotherapy in pretreated patients with metastatic renal cell carcinoma: the multicenter phase II NIVES study. Eur Urol Oncol 2022; 5: 671–679

[45] Giudice GC , Maffezzoli M , Testi I et al. Management of oligometastatic and oligoprogressive renal cell carcinoma: an updated review. Expert Rev Anticancer Ther 2025; 25: 201–213 10.1080/14737140.2025.2464929 39930832

[46] Blackmur J , van der Mijn JCK , Warren A et al. Assessing pathological response to neoadjuvant therapy in renal cell carcinoma: a systematic review and guidelines for sampling and reporting standards from the international Neoadjuvant kidney cancer consortium. Lancet Oncol 2025; 26: e536–e546 41038202 10.1016/S1470-2045(25)00345-6

[47] Anderson AC , Ho J , Hall ET et al. Focal therapy for oligometastatic and oligoprogressive renal cell carcinoma: a narrative review. Future Oncol 2024; 20: 2573–2588 39258792 10.1080/14796694.2024.2389769 PMC11534104

[48] Stenman M , Sinclair G , Paavola P , Wersäll P , Harmenberg U , Lindskog M . Overall survival after stereotactic radiotherapy or surgical metastasectomy in oligometastatic renal cell carcinoma patients treated at two Swedish centres 2005–2014. Radiother Oncol 2018; 127: 501–506 29754859 10.1016/j.radonc.2018.04.028

[49] Welch BT , Callstrom MR , Morris JM et al. Feasibility and oncologic control after percutaneous image guided ablation of metastatic renal cell carcinoma. J Urol 2014; 192: 357–363 24631107 10.1016/j.juro.2014.03.006

[50] Liu C , Cao F , Xing W et al. Efficacy of cryoablation combined with sorafenib for the treatment of advanced renal cell carcinoma. Int J Hyperthermia 2019; 36: 220–228 30663911 10.1080/02656736.2018.1556819

[51] Scheipner L , Incesu RB , Morra S et al. Primary tumor ablation in metastatic renal cell carcinoma. Urol Oncol 2025; 43: 332 10.1016/j.urolonc.2024.10.019 39537442

[52] Stewart GD , Klatte T , Cosmai L et al. The multispeciality approach to the management of localised kidney cancer. Lancet 2022; 400: 523–534 35868329 10.1016/S0140-6736(22)01059-5

[53] Soukup T , Stewart GD , Lamb BW . Defining an evidence‐based strategy for streamlining cancer multidisciplinary team meetings. Lancet Oncol 2023; 24: 1061–1063 37797629 10.1016/S1470-2045(23)00440-0

[54] Morgan RL , Camidge DR . Reviewing RECIST in the era of prolonged and targeted therapy. J Thorac Oncol 2018; 13: 154–164 29113950 10.1016/j.jtho.2017.10.015

[55] Machaalani M , Saliby RM , Zhong C et al. KIM‐1 as a circulating biomarker in metastatic RCC: post‐hoc analysis of JAVELIN renal 101. J Clin Oncol 2025; 43: 594

[56] Verhoeff SR , Oosting SF , Elias SG et al. [89Zr]Zr‐DFO‐girentuximab and [18F]FDG PET/CT to predict watchful waiting duration in patients with metastatic clear‐cell renal cell carcinoma. Clin Cancer Res 2023; 29: 592–601 36394882 10.1158/1078-0432.CCR-22-0921 PMC9890134

[57] Sadaghiani MS , Baskaran S , Gorin MA et al. Utility of PSMA PET/CT in staging and restaging of renal cell carcinoma: a systematic review and Metaanalysis. J Nucl Med 2024; 65: 1007–1012 38782453 10.2967/jnumed.124.267417 PMC11218724

[58] Khene ZE , Bhanvadia R , Tachibana I et al. Predicting recurrence after surgical resection for high‐risk localized renal cell carcinoma: a Radiomics clinical integration approach. J Urol 2025; 214: 296–307 40294223 10.1097/JU.0000000000004588

[59] Smith CG , Moser T , Mouliere F et al. Comprehensive characterization of cell‐free tumor DNA in plasma and urine of patients with renal tumors. Genome Med 2020; 12: 23 32111235 10.1186/s13073-020-00723-8 PMC7048087

[60] Tang C , Sherry AD , Seo A et al. Metastasis‐directed radiotherapy without systemic therapy for oligometastatic clear‐cell renal‐cell carcinoma: primary efficacy analysis of a single‐arm, single‐centre, phase 2 trial. Lancet Oncol 2025; 26: 1289–1299 40915303 10.1016/S1470-2045(25)00380-8

