# Supplementary material for: Oligoprogressive renal cell carcinoma: What is the role of surgery?
Source: BJU Int. 2026 Apr 13;138(2):219–28. doi: 10.1111/bju.70281 (PMC13371407; doi:10.1111/bju.70281)
Supplement: Supplementary file 1 — Table S1 Vocabulary. [file BJU-138-219-s001.docx]

**Supplementary Materials**

**Supplementary Table 1.** Vocabulary

| **Terminology** | **Definition** |
| --- | --- |
| Oligometastatic | Presence of five or fewer metastatic lesions at first cancer diagnosis |
| Synchronous (oligometastatic) | Metastatic disease occurring within a 6-month interval of the primary cancer treatment |
| Metachronous (oligometastatic) | Metastatic disease occurring 6 months after the primary cancer treatment |
| Oligorecurrent | Limited metastases appearing after prior control of the primary tumour |
| Oligoprogressive | Limited disease lesions that acquire resistance to systemic therapy while other lesions remain controlled or continue to respond |
| Oligopersistent | Residual, stable oligometastatic disease following systemic therapy without evidence of progression |

**Two clinical cases of oligoprogressive RCC managed by MDT**

Subject A

A 49y man underwent left radical nephrectomy and adrenalectomy in 2015 for a 9.5 cm left renal mass with associated renal vein tumour thrombus and was found to have a pT3a clear cell RCC (Figure 1). In June 2023, he developed several lesions in the contralateral right kidney, the largest measuring 4 cm and a 7 cm right adrenal metastasis. The patient started a first-line combination therapy with Ipilimumab plus Nivolumab (September 2023), obtaining a partial response with reduction of the adrenal metastasis to 4 cm and stabilisation of the right renal lesions in February 2024. In May 2024, the right adrenal metastasis grew to 6 cm in diameter (oligoprogressive site), and the patient underwent a surgical adrenalectomy. Nivolumab was continued after surgery, with ongoing stability of the residual disease at most recent follow up.

Figure 1: Triple phase abdomen-pelvis CT scan of a) 9.5 cm left renal mass with b) renal vein tumour thrombus. c) 4 cm recurrent lesion in the contralateral right kidney with d) 7 cm right adrenal metastasis. e) stable right renal lesion with 4 cm adrenal metastasis f) 6 cm adrenal metastasis.

Subject B

A 55y man underwent right radical nephrectomy and caval thrombectomy in May 2024 for a 12 cm right renal mass with associated inferior vena cava tumour thrombus and was found to have a pT3b ISUP Grade 4 clear cell RCC (Figure 2). He was considered for adjuvant immunotherapy however the first post-operative scan revealed multiple pulmonary metastatic lesions in the right lung. The patient started a first-line combination therapy with Ipilimumab plus Nivolumab (August 2024), obtaining a significant reduction of lung nodules, other than one enlarging 13 mm right hilar lymph node, which grew further to 21mm (oligoprogressive site). The node showed an FDG avid uptake at the PET scan and was confirmed by endobronchial ultrasound as RCC metastasis. In July 2024, he underwent a lymph node dissection, which demonstrated complete response. Nivolumab has been continued after the surgery.

Figure 2: Triple phase abdomen-pelvis CT scan of a) 12 cm right renal mass with inferior vena cava tumour thrombus. b) multiple pulmonary metastases in the right lung c) 13 mm right hilar lymph node d) increasing right hilar lymph node e) FGD PET avidity of right hilar lymph node.
